# Longitudinal development and tracking of cardiorespiratory fitness from childhood to adolescence

**DOI:** 10.1371/journal.pone.0299941

**Published:** 2024-03-29

**Authors:** Thuridur Helga Ingvarsdottir, Erlingur Johannsson, Vaka Rognvaldsdottir, Runa Sif Stefansdottir, Nanna Yr Arnardottir

**Affiliations:** 1 Center of Sport and Health Sciences, School of Education, University of Iceland, Reykjavik, Iceland; 2 Department of Sport, Food and Natural Sciences, Western Norway University of Applied Sciences, Bergen, Norway; 3 School of Health, Business and Natural Sciences, University of Akureyri, Akureyri, Iceland; Bangor University, UNITED KINGDOM

## Abstract

**Background:**

Cardiorespiratory fitness (CRF) is an important indicator of health in childhood and adolescence but longitudinal studies on the development and tracking of CRF from childhood to adolescence are scarce.

**Objectives:**

The objectives of this study were (1) to assess longitudinal development and track CRF over 10 years from childhood to adolescence, and (2) to examine potential sex differences in the development and tracking of CRF during this period.

**Methods:**

Participants were Icelandic children born in 1999, measured at the age of 7 (n = 190, 106 girls), 9 (n = 163, 95 girls), 15 (n = 239, 134 girls), and 17 (n = 202, 119 girls). CRF was assessed with a maximal cycle ergometer test and expressed as maximal power output (Max W) and maximal power output relative to lean mass (W/kg^LM^). Multilevel regression models were used to study the longitudinal development of CRF, and tracking was assessed with Spearman’s rank correlation, logistic regression, and the percentage of participants remaining in low, moderate, or high CRF categories between measurements.

**Results:**

Max W and W/kg^LM^ increased for both boys and girls up to age 15. Max W plateaued for both boys and girls while W/kg^LM^ plateaued for girls but declined for boys from age 15 to 17. Boys had higher Max W than girls from age 15 and higher W/kg^LM^ from age 9. CRF tracked at low to moderate levels from childhood to adolescence and at high levels in adolescence, with higher values observed for boys than girls.

**Conclusions:**

Age 15 was a critical time point in the development of CRF, with values starting to plateau for girls and decline for boys. The results support early intervention for improved CRF in later years, with interventions targeting all children, regardless of their CRF level.

## Introduction

Cardiorespiratory fitness (CRF) refers to the ability of the respiratory and circulatory systems to transfer oxygen from the atmosphere to the muscles for physical work [1]. CRF has been proven to be an important indicator of health in childhood and adolescence, with high levels of CRF being associated with lower levels of adiposity as well as healthier cardiovascular- and metabolic profiles, both during these years as well as later in life [2–5]. It has been suggested that improving CRF may be an important factor in obesity prevention among youth [4, 6] and, furthermore, that high levels of CRF during childhood and adolescence may counteract the consequences of high levels of fatness during these years [2, 7]. Promoting healthy CRF during childhood and adolescence is, therefore, of great importance for youths’ current and future health.

Longitudinal studies on the development of CRF with chronological age from childhood to adolescence are scarce, especially those covering a wide age range from early childhood to late adolescence [8]. A synthesis of both cross-sectional and longitudinal studies revealed that CRF, when expressed in absolute terms as peak oxygen uptake (peak VO_2_), increases with chronological age from childhood to adolescence, although values seem to level off around the age of 14 for girls [9]. Studies have constantly shown higher absolute values of CRF for boys than girls during childhood and adolescence, with increasing sex differences with age [9–11]. As absolute values of CRF are highly correlated with body size [9], the effect of body size must be accounted for when studying CRF during growth. Lean body mass is considered the predominant influence on the development of CRF during growth [12] and it has been argued that dividing absolute values of CRF by kg of lean mass is therefore appropriate to account for the effect of body size on CRF [13, 14]. Very few studies have examined the longitudinal development of CRF from childhood to adolescence when scaled with lean mass. Existing studies have revealed persistent sex differences in CRF after scaling with lean mass, though results regarding the developmental pattern of lean-mass-related CRF from childhood to adolescence are inconsistent [12, 15–17].

When studying the longitudinal development of CRF, attention should be given to how well it tracks within the group over time. Tracking refers to the individual’s maintenance of their relative rank within the group over time and the predictability of an earlier measurement on a measurement later in life [18]. Tracking CRF levels from childhood to adolescence provides information on when children’s long-term CRF patterns are established and, consequently, provides valuable information for when preventive actions that aim to increase CRF should be implemented [16]. If tracking between two measurement points is found to be high, it indicates that children tend to maintain their rank order over time, meaning that those who have high CRF relative to others in the group at earlier measurements are likely to still have high CRF relative to the others at later measurements. A high degree of tracking would support early intervention for improving CRF, as the benefits of the intervention would be expected to sustain and carry over into later years. Overall, studies have found CRF to track at weak to moderate levels during childhood and adolescence, with tracking values dependent on age, sex, and duration of follow-up [19]. However, very few studies have tracked CRF in the same group of participants at multiple time points during the transition from childhood to adolescence [16, 20–22].

The aims of this study were (1) to assess longitudinal development and track CRF over 10 years from childhood to adolescence, and (2) to examine potential sex differences in the development and tracking of CRF during this period.

## Methods

### Study design and participants

**Fig 1:**
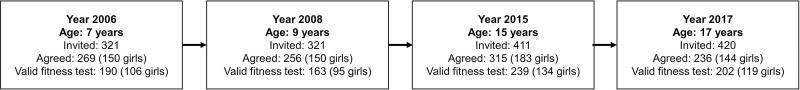
Flow chart describing the study participants.

Six elementary schools in Reykjavík, Iceland, were invited to take part in a cluster-randomized controlled trial called “Lifestyle of seven- and nine-year-olds: Intervention toward better health” in 2006 [23]. All children attending second grade in these schools (born in 1999) were invited to participate (n = 321), of whom 269 (84%) gave informed consent. Baseline measurements were conducted in the fall of 2006, when the participants were 7 years old, and repeated two years later in the fall of 2008 (n = 256, 80%), when they had reached 9 years of age. In 2015, a total of 411 students from the same six schools were invited to participate in a follow-up study called “Health Behavior of Icelandic Youth”. Of the invited participants, 315 agreed to participate (77%) and data collection was carried out from April to June that year. Four hundred and twenty individuals who had been invited to any of the previous measurements (in 2006, 2008, or 2015) were contacted again in 2017 (aged 17) and 236 (56%) agreed to participate. Data collection was conducted from February to April that year. Of the participants who agreed to participate at each measurement point during the study, 190 (71%), 163 (64%), 239 (76%), and 202 (86%) met the criteria for a valid fitness test at the ages of 7, 9, 15, and 17, respectively (Fig 1). In all, 374 participants met the criteria for a valid fitness test at one or more time points during the whole study period. Those who met the criteria for a valid fitness test did not differ statistically from participants who either did not participate in the fitness test or did not meet the criteria for a valid fitness test at any of the four measurement points, except for being slightly taller (138.6 ± 5.1 cm vs. 136.3 ± 6.4 cm, p = 0.004) and having more lean mass (25.0 ± 3.1 vs. 23.1 ± 3.2, p = 0.006) at the 9-year-old measurements, as well as being slightly younger (15.8 ± 0.3 vs. 15.9 ± 0.3, p = 0.014) at the 15-year-old measurements (S1 Table).

Written informed consent was obtained from participants and guardians of underaged participants prior to participation in the study. The study was approved by the National Bioethics Committee as well as the Icelandic Data Protection Authority (VSNb200605002&03 and VSNb2015020013/13.07). Full confidentiality was ensured, and the study was conducted according to the guidance provided in the Declaration of Helsinki. The data was accessed for research purposes in January to September 2023.

### Cardiorespiratory fitness

CRF was measured with a graded maximal cycle ergometer test, using the study protocol of the European Youth Heart Study [24]. The workload on a Monark 829E electronically braked cycle ergometer (Monark Exercise AB, Vansbro, Sweden) was increased every three minutes until the participant reached exhaustion. In 2006 and 2008, the initial and incremental workloads were 20 W for participants weighing less than 30 kg and 25 W for participants weighing 30 kg or more. The test was terminated if the child failed to maintain a pedaling rate over 30 rpm. Heart rate was measured with a Polar M400 heart rate monitor (Polar Vantage, Kempele, Finland) and the criterion for exhaustion was a heart rate of at least 185 beats per minute or subjective evaluation of the researcher of maximal effort. In 2015 and 2017, the initial and incremental workloads were 50 W and 40 W for boys and girls, respectively. Heart rate was measured with a Polar M400 heart rate monitor (Polar Vantage) and participants were asked to rate their perceived exertion on the Borg scale at the end of each stage [25]. The test was terminated if the pedaling rate fell below 40 rpm. At least two out of three of the following criteria had to be fulfilled for the test to be considered maximal: a heart rate of at least 95% of the age-predicted maximum (207 –(0.7 * age) ± 10 beats), perceived exertion of 19–20 on the Borg scale, and the subjective evaluation of the researcher of maximal effort. Maximal power output (Max W) was calculated at all time points using the following formula [26]:

MaxW=Wh+(Wd*t/180)

where Wh represents the workload on the last fully completed stage, Wd represents the increase in workload at each stage, and t represents the time in seconds completed in the unfinished final stage. The maximal power output from this test has been shown to be highly correlated with measured maximal oxygen uptake, suggesting high validity of the test for measuring cardiorespiratory fitness in children and adolescents [24].

CRF was expressed as absolute maximal power output (Max W), as well as maximal power output relative to lean mass (W/kg^LM^) to account for differences in body size [13, 27]. Not all participants underwent DXA measurements, and therefore the number of participants with data on W/kg^LM^ was lower than for Max W.

#### Anthropometrics

Standing height was measured with a stadiometer to the nearest 0.1 cm and body weight was measured to the nearest 0.1 kg using a scale. Lean mass, fat mass, and body fat percentage were measured with dual-energy X-ray absorptiometry (DXA). In 2006 and 2008, the Hologic QDR 4500 pediatric program (Hologic Inc, Marlborough, MA, USA) was used and in 2015 and 2017, the Lunar bone densitometer (GE Healthcare, Wauwatosa, WI, USA) was used. Body fat percentage (BF%) was calculated as fat mass relative to total body mass as measured with DXA (BF% = fat mass/(lean mass + fat mass + bone mineral content)).

### Statistical analyses

Means and standard deviations of all anthropometric variables were calculated at each measurement point by sex. A two-tailed independent samples t-test was used to calculate the mean difference between the sexes. Multilevel regression models were used to study the longitudinal development of Max W and W/kg^LM^ between all measurement points by sex. The multilevel regression models were chosen because of the repeated measurements, the different number of observations per participant, and the clustering of participants within schools. The intercept in the models was allowed to vary across participants and the six different schools, while the year of measurement, sex, and their interaction were treated as fixed effects. A post-hoc test with Bonferroni correction was used to estimate the difference in CRF between measurement points and between the sexes.

Participants with data on W/kg^LM^ at each two measurement points between ages 7, 9, 15, and 17 were ranked and divided into tertiles according to their CRF scores (low, moderate, and high CRF). The tracking of CRF was then assessed in three ways: 1) Spearman’s rank correlation coefficients with two-tailed tests for association were calculated for all age comparisons, for all participants combined as well as by sex. The coefficients range between 0–1 and were classified as suggested by Malina et al. [28]: < 0.3 low, 0.3–0.6 moderate, and > 0.6 good tracking. 2) The percent of participants who remained in the low, moderate, or high CRF category from baseline to follow-up was calculated for all age comparisons. Thirty-three percent of participants would be expected to remain in the same category between measurements by chance, so a percent higher than that expected percent indicates tracking for that variable [18]. This analysis was conducted for the group combined as there wasn’t enough power for sex-specific analysis. 3) Logistic regression was used to calculate odds ratios (ORs), which assessed the odds of having high CRF at follow-up for those who had high CRF at baseline, relative to those who had low or moderate CRF at baseline. All analyses were performed using R (version 4.2.3) and Jamovi (version 2.3.21). The level of significance was set at p < 0.05.

## Results

### Participants’ characteristics

Characteristics of included participants, with respect to sex, are presented in Table 1. Mean scores of all anthropometric variables became higher at every measurement point for both sexes, except for body fat percentage for boys. With respect to sex, girls were slightly older at ages 7, 9, and 17 than boys (p < 0.05). Boys were higher and heavier than girls at ages 15 and 17 (p < 0.001), but no statistical difference in body mass index (BMI) was observed between the sexes (p > 0.05). Boys had more lean mass (p < 0.001) while girls had higher fat mass (p < 0.01) and body fat percentage (p < 0.001) at all measurements, with sex differences being substantially greater at later (15 and 17 years old) than earlier (7 and 9 years old) measurements.

**Table 1:** Characteristics of the participants at each measurement point by sex.

		7 years		9 years		15 years		17 years	
		Girls	Boys	Girls	Boys	Girls	Boys	Girls	Boys
**Age (years)**									
	Mean (SD)	7.4 (0.3)	7.3 (0.3)*	9.4 (0.3)	9.3 (0.3)*	15.8 (0.3)	15.8 (0.3)	17.8 (0.3)	17.6 (0.3)*
	n	106	84	95	68	134	105	119	83
**Weight (kg)**									
	Mean (SD)	26.1 (4.2)	26.6 (4.4)	33.6 (6.1)	34.0 (6.5)	61.2 (9.6)	68.6 (11.3)*	64.3 (10.8)	74.2 (12.3)*
	n	106	84	95	68	134	105	119	83
**Height (cm)**									
	Mean (SD)	126.7 (5.3)	127.5 (5.0)	138.3 (5.3)	138.9 (4.7)	166.2 (5.5)	178.0 (5.7)*	167.9 (5.9)	182.0 (5.9)*
	n	106	83	95	68	134	105	119	83
**BMI (kg/m** ^**2**^ **)**									
	Mean (SD)	16.2 (1.8)	16.3 (1.9)	17.5 (2.4)	17.6 (2.6)	22.1 (3.1)	21.6 (3.2)	22.8 (3.8)	22.4 (3.4)
	n	106	83	95	68	134	105	119	83
**Lean mass (kg)**									
	Mean (SD)	19.2 (2.3)	20.8 (2.2)*	24.2 (3.0)	26.1 (3.0)*	40.0 (4.7)	52.9 (6.4)*	41.6 (4.6)	56.5 (6.8)*
	n	98	79	90	63	132	105	119	83
**Fat mass (kg)**									
	Mean (SD)	7.2 (2.5)	5.9 (2.6)*	9.9 (3.9)	8.1 (4.3)*	18.8 (5.9)	13.1 (7.7)*	20.1 (7.6)	14.7 (8.7)*
	n	98	79	90	63	132	105	119	83
**Body fat (%)**									
	Mean (SD)	25.8 (5.2)	20.5 (5.3)*	27.2 (6.3)	21.8 (6.7)*	30.2 (5.3)	18.4 (7.3)*	30.6 (6.7)	19.0 (7.8)*
	n	98	79	90	63	132	105	119	83

SD: standard deviation; BMI: body mass index.

*Significant difference between the sexes, p < 0.05.

### Development of cardiorespiratory fitness

**Fig 2:**
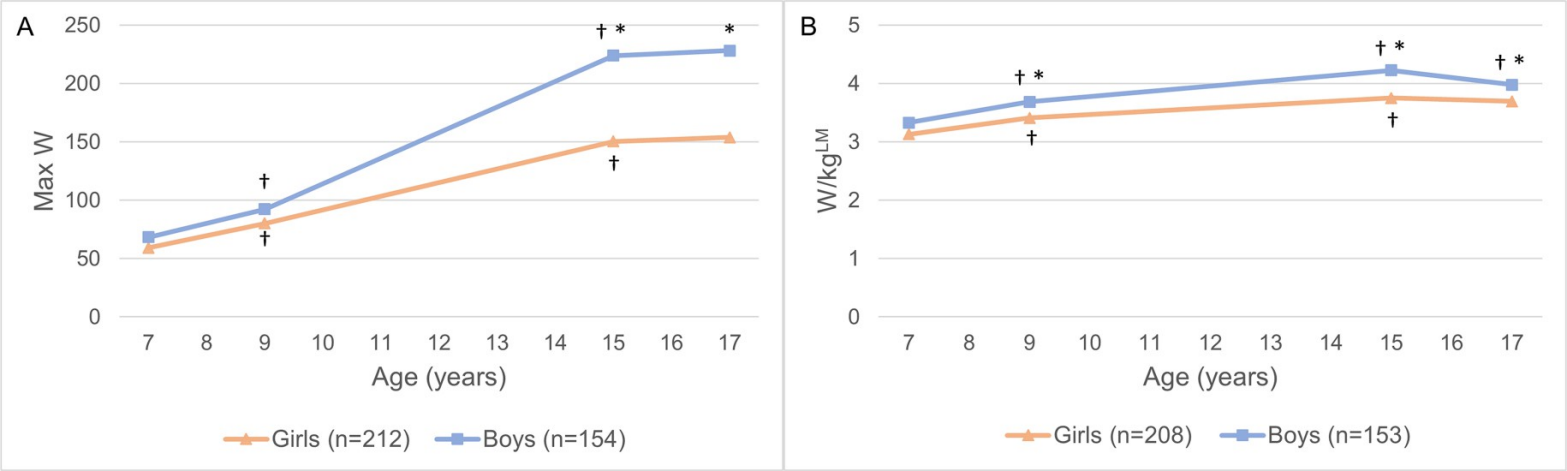
Development of cardiorespiratory fitness. (A) Development of maximal power output (Max W). (B) Development of maximal power output relative to kg lean mass (W/kg^LM^). *Significant difference between the sexes. †Significant difference from previous measurement. P values were obtained with a post-hoc test with Bonferroni correction.

Fig 2 and S2 Table display the results from the multilevel regression models for Max W and W/kg^LM^ at the four measurement points, separately for boys and girls. Information was lacking for 8 participants regarding the school they attended during the study period and these participants were therefore excluded from these analyses. The remaining participants who met the criteria for a valid fitness test at one or more time points during the study period were eligible for the analysis on the development of Max W (n = 366), and those who, additionally, had lean mass measured with a DXA scan were eligible for the analysis on the development of W/kg^LM^ (n = 361).

There were no differences in Max W between girls and boys at ages 7 and 9, but boys had higher Max W than girls at ages 15 and 17. Both boys and girls increased their Max W from age 7 to 15 (p < 0.001) and remained stable from age 15 to 17. No sex differences in W/kg^LM^ were found at age 7; however, boys had higher W/kg^LM^ than girls at ages 9, 15, and 17. Both boys and girls increased their W/kg^LM^ from age 7 to 15 (p < 0.001). Girls’ W/kg^LM^ remained stable from age 15 to 17 while boys decreased their W/kg^LM^ from age 15 to 17 (p = 0.004).

### Tracking of cardiorespiratory fitness

#### Spearman’s rank

Spearman’s rank correlation coefficients, representing the stability of W/kg^LM^ for all age comparisons between ages 7, 9, 15, and 17, are presented in Table 2. For all participants combined, significant correlations were observed between all measurement points, except from age 7 to 17 (p = 0.13). Correlation coefficients were higher for boys than for girls, especially those observed from baseline age 7. The correlations were low to moderate (≤ 0.6) during childhood (from age 7 to 9) and during the transitions from childhood to adolescence (from age 7 or 9 to age 15 or 17), while they were strong (> 0.6) for both boys and girls during adolescence (from age 15 to 17) (Table 2).

**Table 2:** Spearman’s rank correlation coefficients for W/kg^LM^ for all age comparisons between ages 7, 9, 15, and 17.

		Age at follow-up					
		9 years		15 years		17 years	
Age at baseline		n	r	n	r	n	r
**7 years**							
	Girls	78	**0.27** *	51	0.07	49	0.02
	Boys	52	**0.60** ***	47	**0.31** *	40	0.25
	All	130	**0.44** ***	98	**0.27** **	89	0.16
**9 years**							
	Girls			46	0.25	45	0.20
	Boys			40	0.28	33	0.28
	All			86	**0.38** ***	78	0.29*
**15 years**							
	Girls					73	**0.76** ***
	Boys					59	**0.78** ***
	All					132	**0.79** ***

*p < 0.05

**p < 0.01

***p < 0.001

Bold values represent a significant correlation at p < 0.05.

#### Cardiorespiratory fitness categories

**Fig 3:**
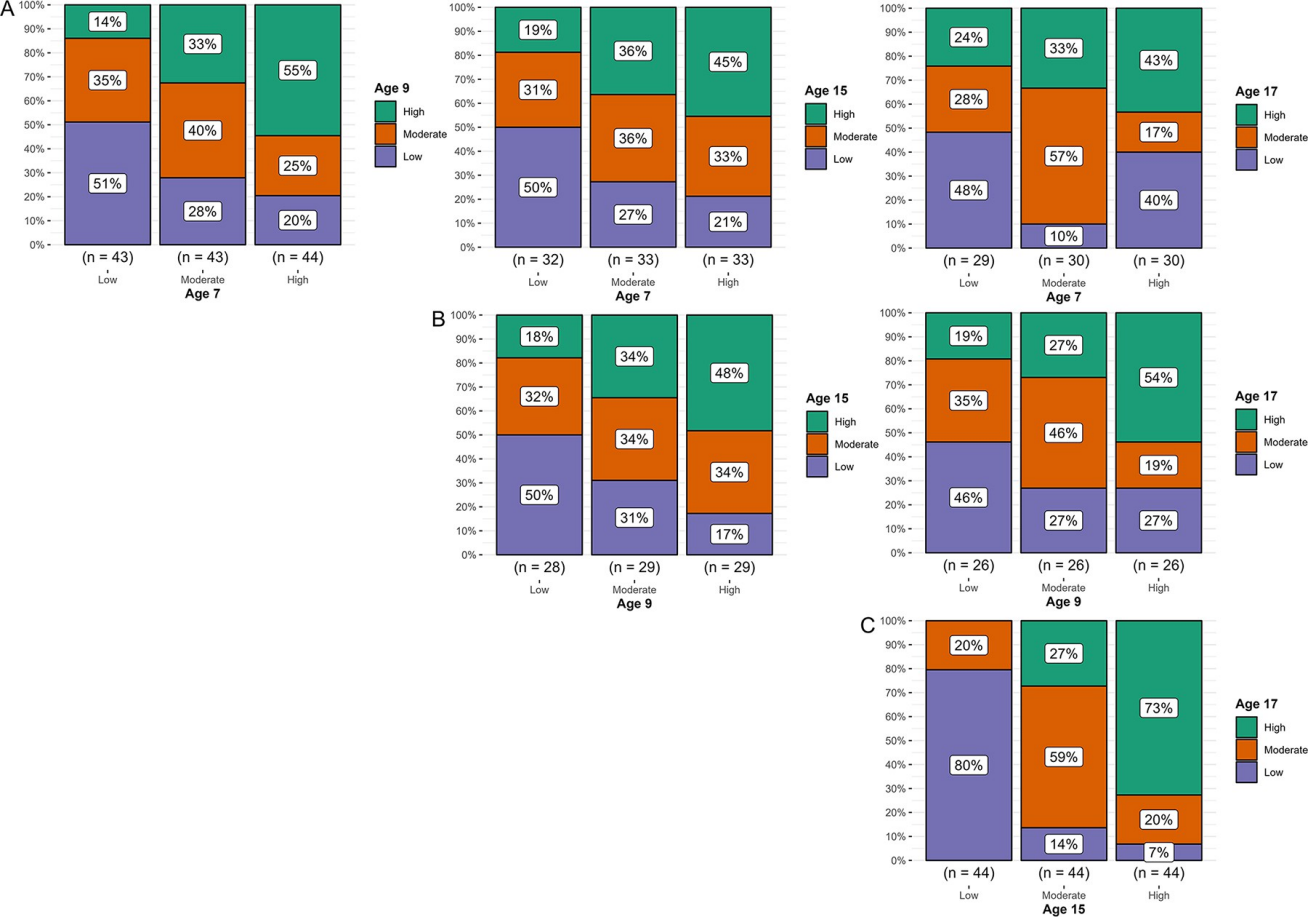
Percent of participants remaining in the low, moderate, or high category for W/kg^LM^. (A) From baseline age 7. (B) From baseline age 9. (C) From baseline age 15. Each box represents the percent of participants in each category at baseline who remained in the same category at follow-up.

The percent of participants staying in each CRF category between each two measurement points are displayed in Fig 3. The percent of participants remaining in the low, moderate, or high category exceeded 33% for all age comparisons, indicating some degree of tracking between all measurement points during the study. Around 46–51% of participants who had low CRF at age 7 or 9 still had low CRF at later measurements, and 43–55% of participants who had high CRF at age 7 or 9 still had high CRF at later measurements (Fig 3A and 3B). The highest degree of tracking was observed between ages 15 and 17, as 80% of participants remained in the low category and 73% remained in the high category from age 15 to age 17 (Fig 3C).

The odds of having high CRF at age 9 were significantly increased for those who had high CRF at age 7 compared to those who had moderate or low CRF at age 7 (p < 0.05) (Table 3). When looking at age comparisons across the transition from childhood to adolescence for all participants combined, significant ORs were observed between ages 9 and 15 (p = 0.045) and 9 and 17 (p = 0.01), but not between ages 7 and 15 (p = 0.08) or 7 and 17 (p = 0.17). The highest ORs were observed in adolescence (between ages 15 and 17), for girls, and boys, as well as for all participants combined. Higher ORs were observed for boys than girls for all age comparisons.

**Table 3:** Odds for having high CRF at follow-up for those with high CRF at baseline, relative to those with low or moderate CRF at baseline, for all age comparisons between ages 7, 9, 15, and 17.

		Age at follow-up					
		9 years		15 years		17 years	
			High vs. low/moderate		High vs. low/moderate		High vs. low/moderate
Age at baseline		n	OR (95% CI)	n	OR (95% CI)	n	OR (95% CI)
**7 years**							
	Girls	78	**3.0 (1.1–8.1)** *	51	1.7 (0.5–5.7)	49	1.0 (0.3–3.6)
	Boys	52	**6.1 (1.7–21.4)** *	47	2.9 (0.8–10.2)	40	1.7 (0.4–6.5)
	All	130	**4.0 (1.8**–8.6)*	98	2.2 (0.9–5.2)	89	1.9 (0.8–4.7)
**9 years**							
	Girls			46	**4.2 (1.2–15.5)** *	45	1.6 (0.4–5.7)
	Boys			40	**7.6 (1.7–32.7)** *	33	4.1 (0.9–19.2)
	All			86	**2.6 (1.0–6.7)** *	78	**3.9 (1.4–10.6)** *
**15 years**							
	Girls					73	**10.6 (3.4–33.0)** *
	Boys					59	**20.4 (5.1–81.1)** *
	All					132	**16.9 (6.9–41.6)** *

OR: odds ratio assessing the odds for being in the high category for W/kg^LM^ at follow-up for those who were in the high category at baseline, relative to those who were in the low or moderate category at baseline.

*p < 0.05

Bold values represent significant ORs at p < 0.05.

## Discussion

This study examined the longitudinal development and tracking of CRF during the transition from childhood to adolescence and assessed if there were any sex differences in the development or tracking of CRF during this period. The results showed that cardiorespiratory fitness, both absolute (Max W) and relative (W/kg^LM^), increased for both boys and girls from age 7 to age 15. Max W was stable from age 15 to 17 for both boys and girls, while W/kg^LM^ was stable for girls but declined for boys from age 15 to 17. Boys had higher Max W than girls from age 15 and higher W/kg^LM^ from age 9. W/kg^LM^ tracked at low to moderate levels in childhood and from childhood to adolescence, and at high levels in adolescence, with a higher degree of tracking observed for boys than girls.

### Development of cardiorespiratory fitness

The CRF levels of the children in our study are similar to those observed for 9- and 15-year-old children in other studies using the same study protocol for measuring CRF [29, 30]. The developmental pattern of Max W for girls observed in this study is also in line with other longitudinal studies that show increasing absolute values of CRF for girls from childhood until around age 14 [9–11]. However, longitudinal studies have, in general, shown absolute values of CRF for boys to continue to increase through adolescence until around age 17 [9–11], while they plateaued at age 15 in our study.

Studies have found lean mass to be the predominant influence on peak VO_2_ during growth and maturation, as the growth and development of active muscle mass both increase the utilization of oxygen in the muscles and increase the delivery of oxygen to the muscles [12]. When the effect of lean mass had been accounted for in our study (W/kg^LM^), CRF increased for both boys and girls up to age 15. For girls, W/kg^LM^ leveled off between ages 15 and 17, which is the time when they have, on average, reached the end of growth and pubertal development. Boys, however, generally hit puberty later than girls and their pubertal development is marked by an increase in lean mass at a much higher rate and for a longer time than for girls [31–33]. Our data showed that absolute CRF plateaued between ages 15 and 17 in boys, despite continuing increases in their lean mass, resulting in declined lean-mass-related CRF during this period.

Why boys’ absolute CRF (Max W) plateaued and their relative CRF (W/kg^LM^) declined in adolescence is not easily discernible. Studies have found that physical activity levels decrease markedly during adolescence [34]. However, as habitual physical activity of children and adolescents rarely reaches the intensity and duration required to improve CRF [35], it is uncertain how much physical activity contributes to CRF levels during those years [36, 37]. Study results based on data from the same cohort as our study showed a 13.1% decline in objectively measured habitual physical activity from age 15 to 17 [38] as well as a decrease in sport participation from 69.1% at age 15 to 50.3% at age 17 [39]. However, the results were not stratified by sex, so it is unknown if the decrease was different for boys than for girls. An increase in average screen time has also been reported in the same cohort from age 15 to 17, though the increase was only significant for girls (from 5.4 to 6.4 hours per day), but not for boys (from 6.0 to 6.4 hours per day) [40].

Other longitudinal studies on the developmental pattern of lean-mass-related CRF from childhood to adolescence are very few. Some studies have reported a stable pattern for boys but a decline for girls [16, 17], while others reported an increase for boys but a stable pattern for girls [15]. A study that used multilevel modeling found an increase in peak VO_2_ with age from 10 to 18 years for both boys and girls after accounting for the effect of fat-free mass, with the effect of age being smaller in girls than in boys [12]. Different methods for measuring and analyzing development in CRF, different ways of measuring lean mass, as well as different expressions of CRF, make comparisons between these studies difficult and may contribute to the discrepancies shown in the results.

Our data showed that boys’ absolute CRF levels were higher than those of girls in adolescence, and the sex differences persisted even after accounting for lean mass, which is in line with other studies [9, 15–17, 41]. The reason for boys having higher CRF values than girls, even when differences in body composition are taken into account, is not readily apparent. Studies on physiological explanations for the sex differences in CRF, including cardiac size and function, have yielded contradictory results [42, 43]. Differences in hemoglobin concentration may serve as a contributing factor in adolescence, as hemoglobin concentration increases in boys during puberty, increasing their oxygen-carrying capacity, and reaches values of about 10% higher than that in girls by late puberty [36]. Differences in physical activity levels between the sexes have also been proposed as a contributing factor, as boys are generally more physically active than girls [44, 45], with sex differences being more pronounced for activity at vigorous intensity [35].

### Tracking of cardiorespiratory fitness

The low to moderate degree of tracking of CRF from childhood to adolescence observed in our study is in line with other studies [16, 19–22]. What makes our study special is the analysis of all age comparisons from four measurement points covering a ten-year age range throughout childhood and adolescence. When the sexes were analyzed separately, higher tracking was observed for boys than for girls, indicating that boys’ CRF levels are more stable throughout childhood and adolescence than those of girls. Other studies have also found CRF to track better among boys than girls during childhood and adolescence [16, 21, 46–49]. This has also been found for physical activity [50] and it might be that a higher level of physical activity among boys compared to girls [44, 45] coupled with higher stability of physical activity within the group, could contribute to a higher degree of tracking of CRF for boys during this period. Other factors that have been found to influence the tracking of CRF in youth are individual differences in growth and maturation [19]. Even though we scaled CRF with lean mass, we may not have captured all aspects of biological maturation that affect CRF and its tracking. Consequently, it may be that variability in the timing and tempo of biological maturation was greater among the girls than the boys in our study, resulting in lower stability in their CRF compared to the boys.

This study showed that around 50% of participants who had low CRF at age 7 or 9 still had low CRF compared to others in the group at later ages. However, this also indicates that around 50% of children who had low CRF in childhood increased their CRF level compared to others in the group when they reached adolescence. Around 20% even increased their CRF level from low to high during this period. This means that CRF levels of children are not strongly determined and can be improved at these ages. Tracking was similar for the low and high CRF groups, meaning that children with high CRF in childhood were just as likely to decrease their CRF when they aged as children with low CRF were to increase their CRF (Fig 3). Actions that aim to promote healthy CRF during youth should, therefore, emphasize increasing the CRF level of those with low CRF, while also maintaining the CRF level of those with high CRF.

Our results showed a high degree of tracking between ages 15 and 17. This was not only due to the relatively short time interval between the measurements, as tracking was much higher compared to an equally sized time interval in childhood (age 7 to 9). This indicates that at age 15, CRF levels had become highly stable within the group: those who had a high fitness level at age 15 were very likely to still have a high fitness level at age 17, while those who had a low fitness level at age 15 were also very likely to have a low fitness level at age 17 (Fig 3C and Table 3). A recent meta-analysis showed that CRF continues to track at moderate levels from adolescence into adulthood, suggesting that improved CRF in adolescence continues to be beneficial for CRF levels and may help improve health in adulthood [51].

Taken together, the results suggest that when applying an intervention to improve CRF before the age of 7, the effects may last up to adolescence for boys, but this is unlikely to be the case for girls. Improving CRF before the age of 9 is, however, more likely to affect CRF levels at the age of 15 and 17 for both sexes. In adolescence, the results suggest high stability in CRF, indicating that improving CRF before the age of 15 should result in enhanced CRF at the age of 17.

### Strengths and limitations

The strength of the study is the longitudinal design and the four measurements over a wide age range, covering the transition from childhood to late adolescence. Another strength is the high-quality cycle ergometer test that measured cardiorespiratory fitness and the objective measurements of body composition with a DXA scan, enabling the expression of CRF relative to lean mass. In addition, the study cohort included all children from six elementary schools in the capital area of Iceland, which represents a relatively large number of all children born in 1999 and living in Iceland during the time of measurements (around 4,200–4,300 children [52–54]).

There are also some limitations that need to be accounted for when interpreting the results. Peak VO_2_ was not measured, which is considered the gold standard for measuring CRF in youth [36]. Studies have, though, found a high correlation between maximal power output and measured peak VO_2_ in these age groups [24]. Also, pubertal status was not assessed, although by expressing CRF relative to lean mass we may have accounted for many of the changes in absolute CRF that are related to maturation [12]. Another limitation of the study is that the validity of CRF measures for children younger than 8 years has been questioned, as young children might have difficulty reaching maximal effort during an exercise test [9]. However, the protocol for the cycle ergometer test used in this study included both objective and subjective criteria for maximal effort and data were not included unless participants fulfilled the criteria. For the tracking analysis, participants were divided into tertiles to detect stability within CRF categories and odds of being in the high category based on earlier measurements. Participants could, though, have been moving within the categories without it having impacted the tracking coefficients, while a minor movement between the borders would have had an impact on the coefficients [18]. The relatively low number of participants with valid CRF data also limited the ability to study sex differences in the tracking of CRF for the tertile-divided data. The sample was, in addition, quite homogenous, which might limit the generalizability of the findings. Future studies should include a larger sample size from heterogeneous populations to enhance understanding of the development and tracking of CRF during youth and how it differs by sex. In addition, future studies should assess potential mechanisms of the development and tracking of CRF during this period and whether the effects of different mechanisms vary by sex.

## Conclusions

Our data showed that age 15 was a critical time point in the development of CRF, with values starting to plateau for girls and decline for boys. The results also revealed that CRF tracked at high levels from age 15 and at low- to moderate levels from age 7 and 9, indicating that improving CRF at an early age may result in improved CRF in later years. Our results may be useful for policymakers and health professionals in targeting interventions for the promotion of healthy CRF, as they support early intervention for improved CRF later in life, with interventions targeting all children, regardless of their current CRF level.

## Supporting information

S1 Table Participants who met the criteria for a valid fitness test at a given measurement (Fitness data) vs. those who either did not participate in the fitness test or did not meet the criteria for a valid fitness test (No fitness data) at that measurement. (DOCX)

S2 Table Estimated marginal means and 95% confidence intervals from multilevel regression models for maximal power output (Max W) and maximal power output relative to kg lean mass (W/kg^LM^) at ages 7, 9, 15, and 17, separately for girls and boys. (DOCX)

S1 Data (CSV)
