# Supplementary material for: Longitudinal development and tracking of cardiorespiratory fitness from childhood to adolescence
Source: PLoS One. 2024 Mar 29;19(3):e0299941. doi: 10.1371/journal.pone.0299941 (PMC10980206; doi:10.1371/journal.pone.0299941)
Supplement: S1 Table — (DOCX) [file pone.0299941.s001.docx]

**S1 Table.** **Participants who met the criteria for a valid fitness test at a given measurement (Fitness data) vs. those who either did not participate in the fitness test or did not meet the criteria for a valid fitness test (No fitness data) at that measurement.**

|  |  | **7 years** | | **9 years** | | **15 years** | | | **17 years** | |
| --- | --- | --- | --- | --- | --- | --- | --- | --- | --- | --- |
|  |  | **No fitness data** | **Fitness data** | **No fitness data** | **Fitness data** | **No fitness data** | **Fitness data** | | **No fitness data** | **Fitness data** |
| **Girls** | | | | | | | |  |  |  |
|  | n (%) | 44 (55.7%) | 106 (55.8%) | 55 (59.1%) | 95 (58.3%) | 49 (64.5%) | 134 (56.1%) | | 25 (73.5%) | 119 (58.9%) |
| **Age (years)** | | | | | | | |  |  |  |
|  | Mean (SD) | 7.3 (0.3) | 7.3 (0.3) | 9.3 (0.3) | 9.3 (0.3) | 15.9 (0.3) | 15.8 (0.3)^*^ | | 17.7 (0.3) | 17.7 (0.3) |
|  | n | 79 | 190 | 92 | 163 | 76 | 239 | | 34 | 202 |
| **Weight (kg)** | | | | | | | |  |  |  |
|  | Mean (SD) | 26.4 (4.7) | 26.3 (4.3) | 32.9 (6.8) | 33.8 (6.2) | 65.6 (11.8) | 64.5 (11.0) | | 67.2 (15.3) | 68.4 (12.4) |
|  | n | 77 | 190 | 92 | 163 | 67 | 239 | | 34 | 202 |
| **Height (cm)** | | | | | | | |  |  |  |
|  | Mean (SD) | 126.9 (5.6) | 127.1 (5.2) | 136.3 (6.4) | 138.6 (5.1)^*^ | 171.8 (7.9) | 171.4 (8.1) | | 171.0 (8.1) | 173.7 (9.1) |
|  | n | 77 | 189 | 92 | 163 | 67 | 239 | | 34 | 202 |
| **BMI (kg/m^2^)** | | | | | | | |  |  |  |
|  | Mean (SD) | 16.3 (2.0) | 16.2 (1.9) | 17.6 (2.6) | 17.5 (2.5) | 22.2 (3.4) | 21.9 (3.1) | | 22.9 (4.4) | 22.6 (3.6) |
|  | n | 77 | 189 | 92 | 163 | 67 | 239 | | 34 | 202 |
| **Lean mass (kg)** | | | | | | | |  |  |  |
|  | Mean (SD) | 20.3 (2.8) | 19.9 (2.4) | 23.1 (3.2) | 25.0 (3.1)^*^ | 45.1 (7.7) | 45.7 (8.5) | | 45.1 (9.5) | 47.7 (9.2) |
|  | n | 34 | 177 | 27 | 153 | 70 | 237 | | 34 | 202 |
| **Fat mass (kg)** | | | | | | | |  |  |  |
|  | Mean (SD) | 6.8 (2.8) | 6.6 (2.6) | 8.2 (3.4) | 9.2 (4.1) | 18.2 (8.7) | 16.3 (7.3) | | 19.4 (11.8) | 17.9 (8.5) |
|  | n | 34 | 177 | 27 | 153 | 70 | 237 | | 34 | 202 |
| **Body fat (%)** | | | | | | | |  |  |  |
|  | Mean (SD) | 23.8 (7.0) | 23.4 (5.8) | 24.7 (6.6) | 25.0 (7.0) | 26.9 (9.3) | 24.9 (8.6) | | 27.9 (10.7) | 25.8 (9.1) |
|  | n | 34 | 177 | 27 | 153 | 70 | 237 | | 34 | 202 |
| SD: standard deviation; BMI, body mass index.  ^*^Significant difference between groups, p < 0.05. | | | | | | | | | | |
