# Supplementary material for: Longitudinal development and tracking of cardiorespiratory fitness from childhood to adolescence
Source: PLoS One. 2024 Mar 29;19(3):e0299941. doi: 10.1371/journal.pone.0299941 (PMC10980206; doi:10.1371/journal.pone.0299941)
Supplement: S2 Table — (DOCX) [file pone.0299941.s002.docx]

**S2 Table.** **Estimated marginal means and 95% confidence intervals from multilevel regression models for maximal power output (Max W) and maximal power output relative to kg lean mass (W/kg^LM^) at ages 7, 9, 15, and 17, separately for girls and boys.**

|  | **Max W** | | | **W/kg^LM^** | | |
| --- | --- | --- | --- | --- | --- | --- |
|  | **Girls (n = 212)** | **Boys (n = 154)** |  | **Girls (n = 208)** | **Boys (n = 153)** |  |
| **Age (years)** | **Mean**  **(95% CI)** | **Mean**  **(95% CI)** | **p^a^** | **Mean**  **(95% CI)** | **Mean**  **(95% CI)** | **p^a^** |
| **7** | 59.1  (54.1-64.0) | 68.3  (62.8-73.9) | 0.43 | 3.13  (3.02-3.23) | 3.33  (3.21-3.44) | 0.24 |
| **9** | 80.0^†^  (74.9-85.2) | 92.3^†^  (86.3-98.4) | 0.07 | 3.41^†^  (3.30-3.52) | 3.69^†^  (3.56-3.82) | **0.02** |
| **15** | 150.4^†^  (145.9-154.9) | 223.8^†^  (218.7-228.9) | **<0.001** | 3.75^†^  (3.65-3.85) | 4.23^†^  (4.12-4.33) | **<0.001** |
| **17** | 153.7  (149.0-158.5) | 228.2  (222.5-233.9) | **<0.001** | 3.69  (3.59-3.80) | 3.98^†^  (3.86-4.10) | **0.003** |
| ^a^p value from a post-hoc test with Bonferroni correction, girls vs. boys. Bold values represent significant difference at p < 0.05.  ^†^Significant difference from previous measurement according to post-hoc test with Bonferroni correction, p < 0.05. | | | | | | |
